# Effect of dipeptide on intestinal peptide transporter 1 gene expression: An evaluation using primary cultured chicken intestinal epithelial cells

**DOI:** 10.1111/asj.13604

**Published:** 2021-07-26

**Authors:** Yukako Tokutake, Marcin Taciak, Kan Sato, Masaaki Toyomizu, Motoi Kikusato

**Affiliations:** ^1^ Animal Nutrition, Life Sciences, Graduate School of Agricultural Science Tohoku University Sendai Japan; ^2^ The Kielanowski Institute of Animal Physiology and Nutrition Polish Academy of Sciences Jabłonna Poland

**Keywords:** CHGA, enterocytes, MUC2, two‐dimensional monolayer culture using collagen gel, VIL1

## Abstract

Peptide transporter 1 (PepT1) is a transporter responsible for absorbing dipeptide and tripeptide in enterocytes and is upregulated by dipeptide in mammals. It has not been certain whether intestinal PepT1 expression is responsive to dipeptides in chickens because of the lack of in vitro study using the cultured enterocytes. This study established a primary culture model of chicken intestinal epithelial cells (IECs) in two‐dimensional monolayer culture using collagen gel by which the response of chicken PepT1 gene expression to dipeptide stimuli was evaluated. The cultured chicken IECs showed the epithelial‐like morphology attached in a patch‐manner and exhibited positive expression of cytokeratin and epithelial cadherin, specific marker proteins of epithelial cells. Moreover, the chicken IECs exhibited the gene expression of intestinal cell type‐specific marker, villin1, mucin 2, and chromogranin A, suggesting that the cultured IECs were composed of enterocytes as well as goblet and enteroendocrine cells. PepT1 gene expression was significantly upregulated by synthetic dipeptide, glycyl‐l‐glutamine, in the cultured IECs. From the results, we herein suggested that dipeptide is a factor upregulating PepT1 gene expression in chicken IECs.

## 1 INTRODUCTION

Peptide transporter‐1 (Pept1, *SLC15A1*) is located in the apical membrane of enterocytes. Pept1 has a high capacity to absorb peptides, which exhibits a broad substrate‐specificity, >400 different dipeptides and >8,000 tripeptides (Wang et al., 2017). Several studies using mammals have demonstrated that intestinal PepT1 expression and dipeptide uptake increased by feeding a high‐protein diet (Erickson et al., 1995; Ferraris et al., 1988). In chicken, intestinal PepT1 gene expression has been reported to be increased by dietary protein quality and levels as well as feed restriction (Chen et al., 2005; Gilbert et al., 2008; Osmanyan et al., 2018) and with the embryonic days and growth (Chen et al., 2005; Miska et al., 2014; Speier et al., 2012). From the findings, it could be suggested that PepT1 plays a vital role in peptide/nitrogen absorption in chicken development and growth.

Effects of dipeptides on intestinal PepT1 expression in mammals have been substantiated by using the intestinal cells cultured with dipeptides, such as glycyl‐l‐glutamine (Gly‐Gln) or glycyl‐sarcosine (Gly‐Sar) (Walker et al., 1998). It has been reported that dipeptides (Gly‐Sar, Gly‐Pro, Gly‐Phe, Met‐Pro, Met‐Lys, Lys‐Lys) did not affect PepT1 promoter activity in chickens; however, the study evaluated the effect using the embryonic fibroblasts (Frazier et al., 2008). Therefore, it remains unclear if dipeptides induce intestinal PepT1 expression in chickens. Thus, the present study aimed to establish primary cultured models of chicken intestinal epithelial cells (IECs) using collagen gels to evaluate the involvement of dipeptides in the upregulation of chicken intestinal PepT1 expression.

The primary chicken IEC culture models have been previously reported (Borrmann et al., 2007; Dimier‐Poisson et al., 2004; Rath et al., 2018; van Deun et al., 2008; Yuan et al., 2015); however, those studies did not evaluate the functionality of differentiated enterocytes. Thus, in the present study, a monolayer on two‐dimensional collagen gel, which based on the report by Kaiser et al. (2017), was used to culture chicken IECs and evaluate the effect of the dipeptide, Gly‐Gln, on the PepT1 expression in the cells.

## 2 MATERIALS AND METHODS

### 2.1 Isolation and culture of chicken IECs

The Animal Care and Use Committee of the Graduate School of Agricultural Science, Tohoku University, approved all procedures (approval number: NOUDOU‐037). Every effort was made to minimize pain and discomfort to the animals.

Chicken fertile eggs (Ross strain, *Gallus gallus domesticus*) were obtained from a commercial hatchery (Matsumoto Poultry Farms & Hatcheries, Miyagi, Japan) and incubated at 38°C/55–65% relative humidity until embryonic age of 18 days. After cleaning the eggshell with 70% (v/v) ethanol, the embryos were quickly removed. The sex of the eggs was not identified. The small intestine was dissected and transferred to Ca^2+^/Mg^2+^ free phosphate‐buffered saline (PBS). After that, mesentery was carefully removed using a scalpel. The intestinal tissues were incised longitudinally and washed gently with ice‐cold PBS five times and then incubated in 20‐mL PBS containing 3‐mM ethylenediaminetetraacetic acid (EDTA) and 0.5‐mM dithiothreitol for 3 min to deactivate the residual protease. The tissues were shredded into several pieces (2–4 mm) on a slide glass and further fragmented by pipetting. The fragments were transferred into a new PBS containing 2‐mM EDTA and incubated for 30 min at 4°C with gentle shaking. The supernatant was discarded and further incubated for 15 min at 4°C in PBS containing 2‐mM EDTA. After shaken by hand vigorously for 15 s, the supernatant containing IECs was collected and filtrated through a 70‐μm Cell Strainer (352350; BD Falcon, Franklin Lakes, NJ, USA). The supernatant was centrifuged at 100 × *g* for 5 min at 4°C. After discarding the supernatant, IECs were resuspended in Dulbecco's Modified Eagle Medium/Ham's Nutrient Mixture F‐12 (DMEM‐F12) (D6421; Sigma‐Aldrich St. Louis, MO, USA). IECs were suspended in the following culture medium, DMEM/F‐12 medium containing 10% fetal bovine serum (FBS) (Gibco, Grand Island, NY, USA), 50 ng/mL human recombinant epidermal growth factor (hEGF) (059‐07873; FUJIFILM Wako Pure Chemical Corporation, Osaka, Japan), 100 ng/mL human noggin (120‐10C; PeproTech, Rocky Hill, NJ, USA), and 1% (v/v) penicillin–streptomycin solution (09367‐34; Nacalai Tesque, Kyoto, Japan).

For the preparation of collagen gel‐coated plate, cold Type‐IA collagen (Cellmatrix Type I‐A; Nitta gelatin, Osaka, Japan) was mixed with culture medium and 20‐mM 4‐(2‐hydroxyethyl)‐1‐piperazineethanesulfonic acid (HEPES). The mixture (pH 7.4) was added to each well of culture plates, and plates were incubated at 37°C for 30 min to reconstruct the wells' collagen. The medium containing isolated IECs was mixed in DMEM/F12 medium and seeded on a collagen gel‐coated plate at 500–1000 tissue fragment/cm^2^. The following plates or slides were used: Nunc™ 24‐well culture dish (# 144530; Thermo Fisher Scientific, San Jose, CA, USA) for morphological observation; TrueLine 6‐well cell culture plate (# TR5000; Nippon Genetics Co.,Ltd., Tokyo, Japan) for total RNA isolation; Nunc™ Lab‐Tek™ II 8‐well Chamber Slide (#154534; Thermo Fisher Scientific) for immunofluorescence.

IECs were incubated under 5% CO_2_ at 37°C, and the medium was discarded and replaced by fresh medium after 2 days of postseeding. IECs were incubated with the culture medium containing 0.4‐mM Gly‐Gln (G0251; Tokyo Chemical Industry, Tokyo, Japan) for 18 h.

### 2.2 Morphological structure and immunostaining of IECs

The morphological structure of the cultured IECs was visualized using Axio Observer A1 microscope (Carl Zeiss, Jena, Germany) equipped with Olympus DP26 digital camera (Olympus, Tokyo, Japan) and imaged using CellSens imaging software (Olympus, Center Valley, PA, USA). IECs were washed with PBS for immunostaining and then fixed with PBS containing 4% (v/v) paraformaldehyde for 15 min. After washing with PBS, IECs were permeabilized with PBS containing 0.1% polyethylene glycol mono‐*p*‐isooctylphenyl ether (Triton®‐X) (12967‐32; Nacalai Tesque) and then blocked with blocking buffer, PBS containing 10% (v/v) goat serum (Gibco) for 30 min. IECs were incubated with antibodies of 2 μg/mL mouse anti‐pan cytokeratin (Novus Biologicals, Littleton, CO, USA) and 20 μg/mL rabbit E‐cadherin antibody (CLOUD‐CLONE CORP., Katy, TX, USA) in blocking buffer overnight at 4°C. IECs were washed with PBS and then incubated with Alexa‐Fluor® 488‐ or 568‐conjugated goat anti‐mouse or ‐rabbit secondary antibodies (1:200 each) (Thermo Fisher Scientific) in PBS for 1 h. The cell nuclei were stained with Hoechst 33342 solution (H342; Dojindo Laboratories, Kumamoto, Japan) for 5 min and then washed with PBS. The fluorescence images were observed using the above systems.

### 2.3 RNA extraction and RT‐PCR

Total RNA was extracted from isolated IECs using the TRIzol reagent (Thermo Fisher Scientific) according to the manufacturer's instructions. RNA purity and concentration were determined using a NanoDrop™ 2000c spectrophotometer (Thermo Fisher Scientific). The total RNA was also extracted from the jejunum of 21‐day‐old male broiler chickens (Ross 308) to determine whether isolated IECs have the intestinal tissues' characters. The isolation was conducted using QuickGene RNA tissue kit S with QuickGene‐Mini‐480 system (Kurabo, Tokyo, Japan) according to the manufacturer's instructions. One microgram of total RNA was reverse transcribed with mixed primers consisted of oligo (dT) and random hexamer into cDNA using M‐MLV Reverse Transcriptase (28025013; Invitrogen, Carlsbad, CA, USA) according to the manufacturer's instructions. The reverse transcription was also performed in the absence of total RNA to prepare the negative control samples that do not contain cDNA. Semiquantitative reverse transcription‐polymerase chain reaction (RT‐PCR) was performed using SapphireAmp® Fast PCR Master Mix (RR350A; TaKaRa Bio, Kusatsu, Shiga, Japan) to analyze intestinal epithelial‐specific marker's gene expressions: enterocyte, villin1 (*VIL1*); goblet cells, mucin 2 (*MUC2*); enteroendocrine cells, chromogranin A (*CHGA*); undifferentiated cells, leucine‐rich repeat‐containing G protein‐coupled receptor 5 (*LGR5*), olfactomedin 4 (*OLFM4*), and prominin 1(*PROM1*). The following reactions were carried out: 94°C for 1 min, followed by 40 cycles of 98°C for 15 s, 58°C for 15 s, and 72°C for 15 s. PCR products were electrophoresed in a 3% agarose gel. The bands were visualized in a trans‐illuminator (UVP Benchtop 2UV; Fisher Scientific, NJ, USA) and pictured using STAGE‐1000 (AMZ System Science, Osaka, Japan). PepT1 gene expression level was determined by quantitative RT‐PCR (RT‐qPCR) using SYBR Premix Ex Taq TM II (RR820S; TaKaRa Bio). The values were normalized to β‐actin expression level and shown as fold changes relative to the values of nontreated cells. The PCR products from each primer pair were subjected to a melting analysis and subsequent agarose gel electrophoresis to confirm amplification specificity. RT‐qPCR was performed using the CFX Connect™ system (Bio‐Rad Laboratories, Hercules, CA, USA), and data were analyzed based on the Pfaffl method. Primer sequences are listed in Table 1.

**TABLE 1:** Primer sequence

Gene symbols	Accession no.	Sequence (5′‐3′)		Product size (bp)
*LGR5*	XM_425441.5	Forward:	TCTCCAGGTCCCTTCAAACC	218
		Reverse:	ACGCTAGCCAGTACTCCACT	
*OLFM4*	NM_001040463.1	Forward:	ACAACGACAGACGTGACTCC	170
		Reverse:	GGAAAGGTGGTATCCGGCAA	
*PROM1*	NM_001291649.1	Forward:	CTGCCAACCAACACTTAACTAGCCA	263
		Reverse:	TTCTCTGATTGCTCCTGCCATTGTC	
*VIL1*	NM_205442.1	Forward:	ATCATCGTGGTGAAGCAGGG	158
		Reverse:	GAGGTGAGCCCTGACACAAG	
*CHGA*	XM_421330.6	Forward:	AGGAGAACAGAGGACCAGGAG	225
		Reverse:	CAACACTACTGCGAGGGAGG	
*MUC2*	NP_001305363.1	Forward:	CAGCGTTAACACAGGGCTTA	94
		Reverse:	GCAGCAGACGTTGATCTCAT	
*PepT1 (SLC15A1)*	NM_204365.1	Forward:	TGAGCAGTGGGCAGAATATGT	289
		Reverse:	ACCCAGTGATGTCAGCAATACC	
*β‐Actin*	NM_205518.1	Forward:	CTGGCACCTAGCACAATGAA	123
		Reverse:	CTGCTTGCTGATCCACATCT	

Abbreviations: CHGA, chromogranin A; LGR5, leucine rich repeat containing G protein‐coupled receptor 5; MUC2, mucin 2; OLFM4, olfactomedin 4; PepT1, peptide transporter 1; PROM1, prominin 1; VIL1, villin1.

### 2.4 Statistical analysis

Based on at least three repeats in each experimental group, all data are represented as the mean ± standard error of the mean (*n* = 3). Statistical significance was determined using Shirley–Williams test for comparisons between groups. *P* < 0.05 was considered statistically significant.

## 3 RESULTS

### 3.1 Morphological characteristics of chicken IECs

**FIGURE 1:**
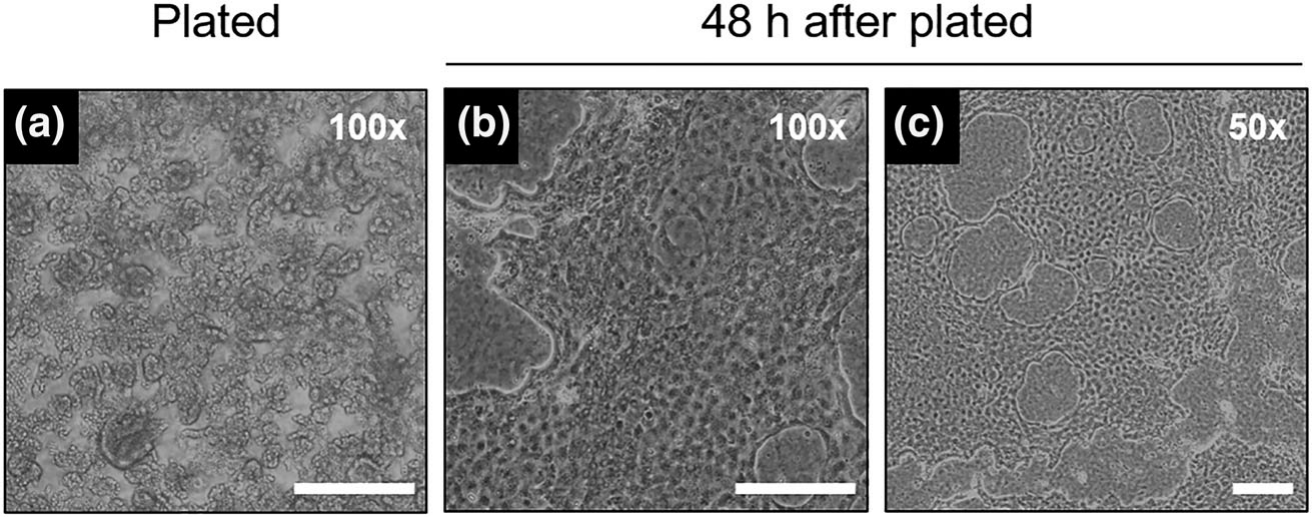
Morphological structure of primary cultured chicken intestinal epithelial cells (IECs), 0‐h (a) and 48‐h postseeding (b) with high magnification (100×), 48‐h postseeding IECs with low magnification (50×) (c). Images were captured under a phase contrast. Scale bars = 200 μm each

A large number of tissue fragments were observed immediately after seeding (Figure 1a). After 48‐h incubation, IECs adhered in a patchy manner and were covered the entire area of the dish surface (Figure 1b,c). Cultured IECs exhibited a cobblestone morphology, a typical structure of epithelial cells, suggesting that the cell population was composed of epithelial cells.

### 3.2 Gene expression of the epithelial‐specific factors in IECs

**FIGURE 2:**
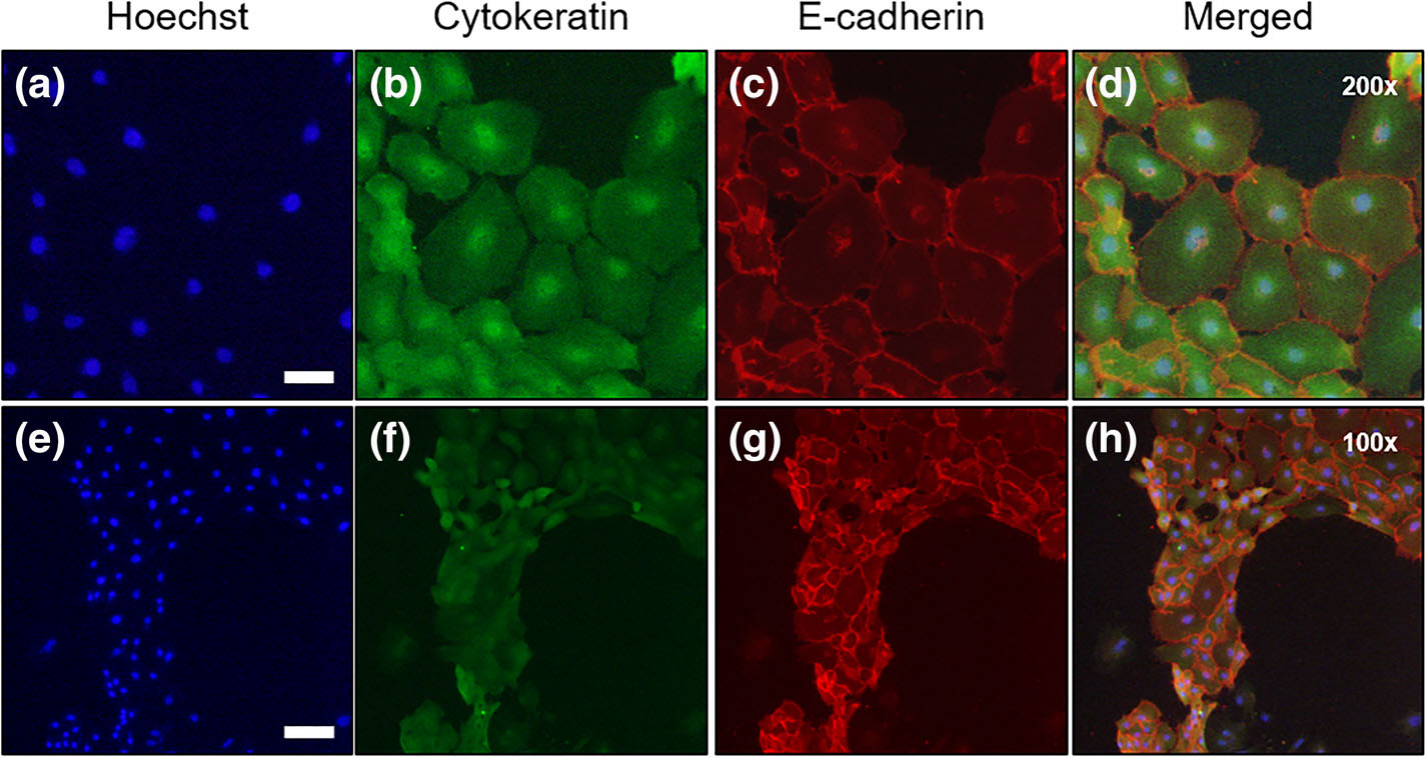
Immunofluorescence staining of epithelial‐specific markers in primary cultured chicken intestinal epithelial cells (IECs). IECs stained with Hoechst 33342 (a,e), cytokeratin (b,f), E‐cadherin (c,g), and merged images (d,h) were visualized with high magnification (200×) (upper) and low magnification (100×) (lower). Scale bars = 50 μm (upper) and 100 μm (lower). The experiment was repeated three times, and the representative data were shown

IECs consist of four major types of cells in the intestinal epithelium: enterocytes, goblet cells, enteroendocrine cells, and Paneth cells. The epithelial cell‐specific markers were evaluated in the cultured chicken IECs. Cytokeratin is a keratin protein explicitly localized in the cytoskeleton of epithelial cells, and most IECs exhibited cytokeratin‐positive (Figure 2b,f). Similarly, E‐cadherin, an intercellular adhesion molecule localized in the membrane surface of epithelial cells, was detected in the cell membrane (Figure 2c,g). As visualized in Figure 2d,h, cytokeratin and E‐cadherin were co‐expressed in the cultured IECs. Based on these lines of evidence, it can be suggested that the cultured chicken IECs in this study have epithelial cell character.

**FIGURE 3:**
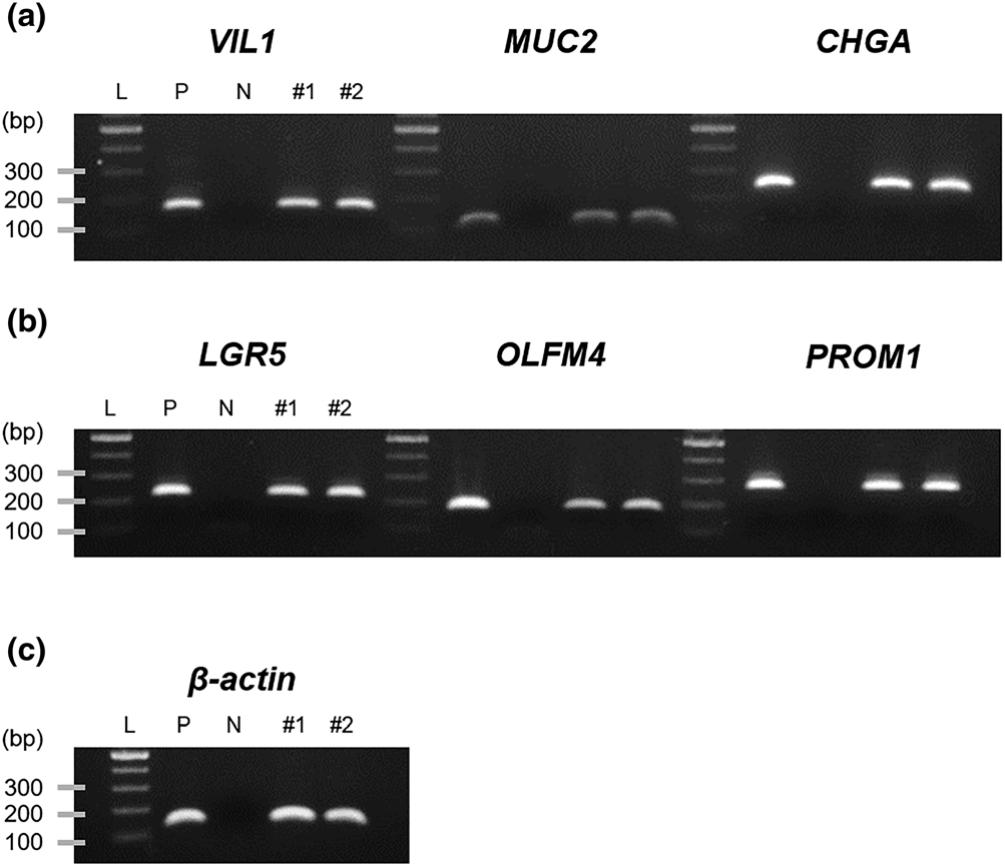
Gene expression of intestinal cell‐specific markers of primary cultured chicken intestinal epithelial cells (IECs). Differentiated enterocytes, *VIL1* (158 bp); goblet cells, *MUC2* (94 bp); enteroendocrine cells, *CHGA* (225 bp) (a); undifferentiated/stem cells, *LGR5*, *OLFM4*, *PROM1* (218, 170, 263 bp) (b); housekeeping gene, *β‐actin* (123 bp) (c). Gene expression was measured by semiquantitative reverse transcription‐polymerase chain reaction (RT‐PCR) and visualized with agarose gel electrophoresis. L, DNA ladder; P, positive control from 21‐day‐old chicken's jejunum; N, a negative control sample without cDNA; #1, 2, samples from cultured IECs. The experiment was repeated three times, and the representative data were shown

The study next evaluated gene expression of the intestinal epithelial‐specific markers in the cultured IECs. As seen in Figure 3a, gene expression of an enterocyte marker, *VIL1*, was similarly detected in the cultured IECs (Lanes #1 and #2) to juvenile chickens' jejunum tissues (Lane “P”). Goblet and endocrine cell‐specific markers, *MUC2* and *CHGA,* were also expressed in both IECs and the jejunum tissues. The results indicate that the cultured IECs contain enterocytes as well as other types of differentiated cells. Meanwhile, the gene expression of undifferentiated cell markers, *LGR5*, *OLFM4*, and *PROM1*, was also found in the IECs (Figure 3b).

### 3.3 The response of PepT1 expression to dipeptide stimulation in chicken‐cultured IECs

The study examined the effects of dipeptide supplementation, Gly‐Gln, on *PepT1* gene expression in cultured chicken IECs. The IECs treated with 0.4‐mM Gly‐Gln exhibited a 2.5‐fold increase (*P* < 0.05) in *PepT1* expression compared with the nontreated cells, whereas the increase was not observed in the IECs treated with 4‐mM Gly‐Gln.

## 4 DISCUSSION

The present study used a monolayer culture model on the collagen gel to culture chicken enterocytes. The collagen gel‐based culture system enabled to attach IECs at a higher density than the collagen‐coated system, which was used in recent reports (Kaiser et al., 2017; Rath et al., 2018). The present study showed that the IECs cultured by the gel‐based system showed positive immunostaining reactions for cytokeratin and E‐adeherin (Figure 2) as well as gene expression of the specific markers of enterocytes, goblet, and endocrine cells (Figure 3). The presence of Paneth cells in avian species has been investigated, whereas lysozyme expression, a biomarker of Paneth cells, was detected in adult birds but not in the embryos (Nile et al., 2004; Wang et al., 2016). The present study also found the gene expression of *LGR5*, *OLFM4*, and *PROM1*, each found in undifferentiated epithelial stem cells (Zhang & Wong, 2018; Zhu et al., 2009). These lines of evidence suggest that the chicken IECs have epithelial cell character and can be used as an in vitro model to evaluate chicken intestine functions. It needs to note that the cultured IECs in the study did not proliferate, and most cells were detached at 4 days of postseeding (data not shown), suggesting that the exogenous growth factors may be required to induce proliferation of the cells.

**FIGURE 4:**
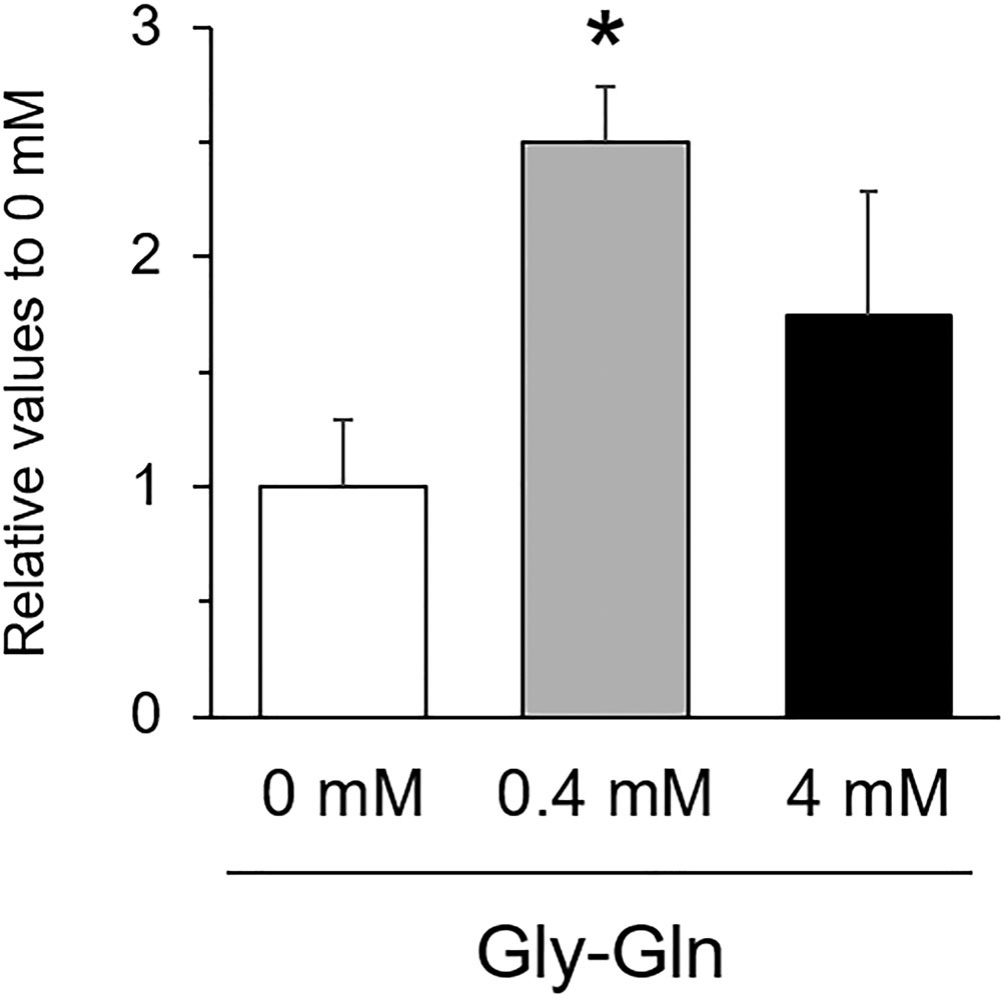
The effect of Gly‐Gln (0.4, 4.0 mM) on PepT1 expression of primary cultured chicken intestinal epithelial cells (IECs). Reverse transcription‐polymerase chain reaction (RT‐qPCR) determined gene expression. Data were shown as means ± S.E. from three individual cell samples, and the values were shown as fold changes relative to the nontreated cells. *P < 0.05

Several studies using mammalian cells have reported that PepT1 promoter activity and transcriptional level were elevated by various dipeptides and amino acids. The supplementation of 4‐mM Gly‐Gln upregulated hPepT1 protein and mRNA levels in Caco‐2 cells (Walker et al., 1998). The present study showed that 0.4‐mM Gly‐Gln supplementation increased PepT1 gene expression in the cultured chicken IECs, while the upregulation was not observed in the cells treated with 4‐mM Gly‐Gln (Figure 4). For this reason, it might be considered that chicken primary IECs are more sensitive to dipeptide stimuli compared with the cell line used in the previous investigation. It has been reported that PepT1 promoter activity in chicken embryo fibroblasts did not respond to dipeptides tested in transfected chicken embryonic fibroblasts (Frazier et al., 2008). Therefore, the present study was the first to demonstrate PepT1 gene expression in response to dipeptide stimuli in chicken enterocytes isolated from the intestinal tissues. Not only dipeptides but also butyric acid and resveratrol have also been shown to increase PepT1 gene expression in cultured mammalian cells (Dalmasso et al., 2008; Jia et al., 2015; Shu et al., 2019). Therefore, the present IEC model could be used to select dietary constituents that can induce PepT1 gene expression and its related nitrogen/peptide absorption in chickens.

In conclusion, the present study demonstrated that primary chicken IECs cultured using the collagen‐gel system have epithelial cell character, and PepT1 gene expression responded to dipeptide stimuli, as with the case with mammalian intestinal cells. It also suggested that the IECs may consist of enterocytes, goblet, and endocrine cells, suggesting that the cells could explore the factors affecting peptide/amino acid transporter and the cell functions in the chicken intestine.

## CONFLICT OF INTEREST

The authors declare no conflict of interest.
